# Acknowledgement to Reviewers of *Membranes* in 2018

**DOI:** 10.3390/membranes9010017

**Published:** 2019-01-17

**Authors:** Membranes Editorial Office

**Affiliations:** MDPI AG, St. Alban-Anlage 66, 4052 Basel, Switzerland

Rigorous peer-review is the corner-stone of high-quality academic publishing. The editorial team greatly appreciates the reviewers who contributed their knowledge and expertise to the journal’s editorial process over the past 12 months. In 2018, a total of 136 papers were published in the journal, with a median time to first decision of 14 days and a median time to publication of 36 days. The editors would like to express their sincere gratitude to the following reviewers for their cooperation and dedication in 2018:
Abejón, RicardoLao, Ka UnAlbo, JonathanLee, ByeongchanAlem, HalimaLee, Eon SooAlique, DavidLee, Jae-SukAnnesini, Maria CristinaLee, KangwonAnsaloni, LucaLee, Kew-HoAnsón Casaos, AlejandroLemma, SolomonArencón, DavidLeón, Gerardo A.Arnusch, ChristopherLin, RijiaAssender, HazelLinke, DirkBabaRao, RavichandarLinkous, Clovis A.Baji, AvinashLipnizki, FrankBallmer-Hofer, KurtLipscomb, GlennBaltus, RuthLiu, ShaominBaumann, StefanLiu, XinfengBella, FedericoLiu, YaodongBellardita, MariannaLlosa Tanco, Margot A.Benavente, JuanaLow, NicholosBiswal, Bishnu PrasadLufrano, FrancescoBiswas, SoumavaLuo, ShuaiBlandin, GaetanLv, KangleBrazinha, CarlaMardilovich, Ivan P.Brinkmann, TorstenMarkondeya Raj, PulugurthaBrown, DavidMarquardt, DrewBrunklaus, GuntherMarracino, PaoloBrunner, AndreasMartínez-Espinosa, Rosa MaríaBulgariu, LauraMartinka, JozefBuonomenna, Maria GiovannaMatsumoto, MichiakiByrne, BernadetteMazzei, RosalindaCalabrò, FrancescoMele, AndreaCalero, CarlesMelidis, ParaschosCamblor, Miguel A.Mendoza-Roca, José AntonioCampos-Martínez, J.Menéndez, MiguelCannilla, CatiaMilčius, DariusCao, YuheMinelli, MatteoCarreon, MoisesMirna, Habuda-StanicCasado-Coterillo, ClaraMoratti, StephenCassano, AlfredoMueller, AnjaCastro Dominguez, BernardoMukherjee, SoumyaCerneaux, SophieNagy, EndreChantana, JakapanNegro, EnricoChen, GeorgeNemestothy, NandorChen, JinhuaNghiem, LongChen, JunNicoletta, Fiore PasqualeChew, John Y.MNijmeijer, ArianChristensen, Morten LykkegaardNishihara, MasamichiChun, YoungpilNulwala, HunaidCicciù, MarcoOchiai, BungoCoelhoso, IsabelOgórek, RafałComas, JoaquimOllila, SamuliCoronas Ceresuela, JoaquínPacheco Tanaka, David AlfredoCortina Pallas, JosePagano, StefanoCosta, Carlos M.Pagliara, StefanoCowan, Matthew GreigPanglisch, StefanCzech, BożenaPaolone, AnnalisaDa’na, EnshirahPark, Chul-hoDarling, SethPatsios, Sotiris I.De Grooth, JorisPatterson, JenniferDe Pinho, Maria NorbertaPeters, ThijsDeimede, ValadoulaPontié, MaximeDell’Agli, GianfrancoPop, OanaD’Errico, GerardinoPouponneau, PierreDi Palma, LucaPulyalina, AlexandraDi Trapani, DanieleQuist-Jensen, Cejna AnnaDong, YingchaoRajca, MariolaDrobek, MartinRana, DipakDrozdov, Aleksey D.Raudino, AntonioDumée, LudovicReshetenko, Tatyana V.Dutournié, PatrickRishpon, JudithEsposito, ElisaRissanou, Anastassia N.Evans, PaulRobert, EricFan, WeizhengRodriguez, NoelFarrugia, BrookeRonen, AvnerFatyeyeva, KaterynaRowley, ChristopherFauzi, IlianaRoy, SagarFerguson, MatthewRóżycki, BartoszFerreira, Diana P.Ruiz-García, AlejandroFievet, PatrickRyi, Shin-KunFonseca, Ana ClotildeSadoway, Donald R.Froeyen, MatheusSadrzadeh, Mohtada Fu, QiangSalinas, SergioFujimoto, CySamhaber, W. M.Fujita, MasahiroSanyal, OishiFun, Lim YeeSarti, Giulio C.Fuoco, AlessioSelyanchyn, RomanFutakuchi, MitsuruSemsarilar, MonaGallucci, FaustoSequeira, CésarGarcía Ivars, JorgeShahid, SalmanGeise, GeoffreyShamsaei, EzzatollahGeißen, Sven-UweShao, LuGeorgopanos, ProkopiosShen, YuexiaoGhalei, BehnamSim, Lee NuangGierszewska, MagdalenaSimari, CataldoGil-Mestres, AdriàSioutopoulos, DimitriosGlüsen, AndreasSirkar, KamaleshGnade, Bruce E.Sledzinska, MariannaGorri, DanielSmiatek, JensGray, StephenSodt, Alexander J.Grizzuti, NinoSpallina, VincenzoGu, XiaodanSrebnik, SimchaHélix-Nielsen, ClausSrinivas, KeerthiHelmi, ArashStuckey, David C.Henderson, Luke C.Szekely, GyorgyHernandez, AntonioTai-Shung Chung, NealHidalgo, A. M.Tarabara, VladHiga, MitsuruTena, AlbertoHinrichs, JörgThakur, Vijay KumarHirota, YuichiroThibault, JulesHong, LiangTiemblo, PilarHong, TaoToikka, AlexanderHou, JingweiTopin, JeremieIbáñez Mendizábal, RaquelTringe, JosephIfelebuegu, AugustineTrovato, FabioIliev, BoyanTsay, Chien-YieIndiveri, CesareTylkowski, BartoszIulianelli, AdolfoVan Sint Annaland, MartinJacak, JarosławVancaeyzeele, CédricJamin, NadègeVictor, BrunoJamróz, PiotrVoyiatzis, GeorgeJansen, JohannesWagner, StefanJørgensen, Mads KoustrupWang, David K.Julien, Christian M.Wang, ZhiweiJung, Kyung-HyeWei, GangKalogiannis, Konstantinos G.Woldemariam, DanielKarakaya, CananWu, BingKaranikola, VasilikiWycisk, RyszardKazuhiro, ShikinakaWyman, IanKelly, Christopher V.Xiang, YuanKentish, SandraXu, TaoKertèsz, SzabolcsXue, JinkaiKim, HyunsungYalcinkaya, FatmaKim, JeongYang, ShaoweiKim, Seok-JhinYin, LanKong, LingxueYoo, Dong JinKononova, Svetlana V.Yoshihiko, SanoKoter, StanislawYu, JaecheulKotsuchibashi, YoheiYu, Tzyy LeonKowalska, EwaZha, ShangwenKruczek, BoguslawZhang, LijunKujawa, JoannaZhang, SuiKujawski, WojciechZhang, XueqingKulozik, UlrichZhang, YanlinKvasha, AndriyZhao, WeifengLade, HarshadZhu, ShanLaguna, O.H.Zuo, JianLaity, Peter R


